# Moth resonant mechanics are tuned to wingbeat frequency and energetic demands

**DOI:** 10.1098/rspb.2024.0317

**Published:** 2024-06-26

**Authors:** Ethan S. Wold, Brett Aiello, Manon Harris, Usama bin Sikandar, James Lynch, Nick Gravish, Simon Sponberg

**Affiliations:** ^1^ School of Biological Sciences, Georgia Institute of Technology, Atlanta, GA 30332, USA; ^2^ School of Natural and Health Sciences, Seton Hill University, Greensburg, PA 15601, USA; ^3^ School of Physics, Georgia Institute of Technology, Atlanta, GA 30332, USA; ^4^ School of Electrical and Computer Engineering, Georgia Institute of Technology, Atlanta, GA 30332, USA; ^5^ Mechanical and Aerospace Engineering, University of California San Diego, San Diego, CA 92161, USA

**Keywords:** moth, flight, resonance, exoskeleton, wing

## Abstract

An insect’s wingbeat frequency is a critical determinant of its flight performance and varies by multiple orders of magnitude across Insecta. Despite potential energetic benefits for an insect that matches its wingbeat frequency to its resonant frequency, recent work has shown that moths may operate off their resonant peak. We hypothesized that across species, wingbeat frequency scales with resonance frequency to maintain favourable energetics, but with an offset in species that use frequency modulation as a means of flight control. The moth superfamily Bombycoidea is ideal for testing this hypothesis because their wingbeat frequencies vary across species by an order of magnitude, despite similar morphology and actuation. We used materials testing, high-speed videography and a model of resonant aerodynamics to determine how components of an insect’s flight apparatus (stiffness, wing inertia, muscle strain and aerodynamics) vary with wingbeat frequency. We find that the resonant frequency of a moth correlates with wingbeat frequency, but resonance curve shape (described by the Weis-Fogh number) and peak location vary within the clade in a way that corresponds to frequency-dependent biomechanical demands. Our results demonstrate that a suite of adaptations in muscle, exoskeleton and wing drive variation in resonant mechanics, reflecting potential constraints on matching wingbeat and resonant frequencies.

## 1. Introduction

Fast cyclic movements are key to many organisms' locomotor performance and have given rise to the convergent evolution of elastic structures that help offset inertial costs of locomotion by storing and releasing energy from cycle to cycle [1–3]. Spring-like structures may also constrain animal performance by making some frequencies of movement less energetically favourable than others. Any system with inertia and elasticity will have a resonant frequency, defined by its mass, spring and damping properties. An animal moving at its resonant frequency theoretically benefits from a larger kinematic output (i.e. limb motion) for the same actuation force input, since relatively small mechanical energy inputs during each cycle compound to create a larger amplitude oscillation than would be possible at a non-resonant frequency [4,5]. Conversely, operation at resonance may be detrimental to manoeuvrability, since an animal seeking to modulate its limb movement must work against a large amount of limb mechanical energy built up over many oscillations [6]. The potential advantages and disadvantages of operating at resonance suggest that animals may balance trade-offs in the diversification of actuation and biomechanics across species with different behavioural and energetic requirements.

**Figure 1.**
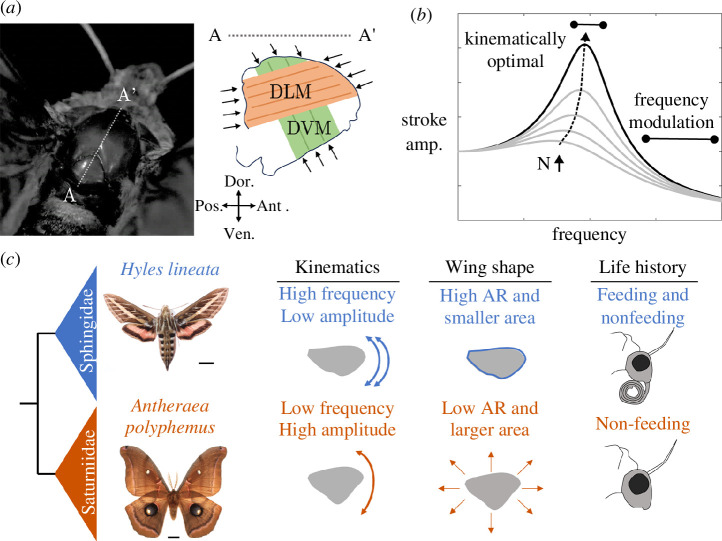
(*a*) Photograph of a hawkmoth thorax with the cross-section along line AA' shown to the right. The downstroke (DLM) and upstroke (DVM) muscles are shown, along with their lines of action. Modified with permission from [4]. (*b*) Generalized resonance curve for a spring-mass damper system, showing regimes of kinematically optimal and more manoeuvrable wingbeat frequencies. Grey curves show the effect of increasing N. (*c*) Hawkmoths (sphingids) and silkmoths (saturniids) are sister families that make up the superfamily Bombycoidea. The two clades exhibit different flight kinematics, wing morphologies (aspect ratio (AR) and wing area) and life-history traits. Modified with permission from Aiello *et al*. 2021 [12].

Resonance tuning may facilitate higher insect wingbeat frequencies, which could help smaller flapping organisms deal with their relatively higher mass-specific energy costs [7–9]. Most insects actuate their wings indirectly, through muscles that attach to the outside of a thin elastic exoskeletal shell [10,11], which deforms to transmit muscle strain to rotational movement of inertial wings via a transmission known as the wing hinge (figure 1*a*). Flying insects move through the air, meaning that aerodynamically useful work done to support body weight is mostly dissipated to the surrounding fluid. As such, flying insects have been modelled as forced oscillators with elasticity, inertia and nonlinear aerodynamic damping—a system with resonant mechanics [4,9,13]. However, operating at resonance may also inhibit rapid frequency modulation of the wing stroke, because modulating frequency requires active muscular work against the movement of the wings and a temporary reduction of stroke amplitude (figure 1*b*). Frequency modulation may be important in agile flyers such as hawkmoths that rapidly manoeuvre in complex aerial environments [6]. Evaluating this trade-off between energy and frequency modulation across insects remains a challenge due to the lack of comparative measurements of spring-wing mechanics under a consistent model of resonance.

The aerodynamic efficiency-agility trade-off implied by resonant wingbeats was pioneered by the work of Weis-Fogh in the mid-20th century, who defined a non-dimensional number (which we refer to as the Weis-Fogh number, N), measuring the ratio of peak inertial to aerodynamic torque over a wing stroke [8,13]. N has a convenient interpretation as the ‘sharpness’ of an insect’s resonant curve (wingbeat amplitude versus wingbeat frequency), analogous to the quality factor (Q) in engineered systems. Higher N implies a larger inertial power requirement relative to aerodynamics, and therefore both a larger potential benefit from elastic energy savings and steeper energetic penalty for off-resonance wingbeats. Lower N implies a larger relative aerodynamic power requirement and a larger fraction of total work done being used to support body weight, but a shallower resonance curve. The Weis-Fogh number provides a convenient non-dimensional value for comparing resonant trade-offs across species and has previously been measured in insects in the range of 1<N<8 [8,13,14].

While there is little question that insects exhibit resonant mechanics [7,8,15,16], direct measurements of resonant wingbeats have been limited to phylogenetically isolated species with disparate methods that complicate cross-species comparisons [4,14,17–19]. Furthermore, without independent, comparative measurements of each flight apparatus component, one cannot distinguish traits that facilitate resonance tuning from traits that counteract resonance tuning due to competing constraints. For example, smaller (faster-flapping) insects actuate large-amplitude wingstrokes with smaller muscle displacements [20], increasing their transmission ratio compared with larger insects (ratio of stroke angle amplitude to muscle displacement amplitude). As we demonstrate later, this in isolation would dramatically lower their resonant frequency [4], contrary to the expectation of resonance tuning. In general, it remains unknown how flight apparatus components scale with wingbeat frequency in closely related species, and whether this variation contributes to the evolutionary tuning of resonant mechanics.

The superfamily of moths Bombycoidea offers an opportunity to comparatively study the biomechanical drivers of insect resonant mechanics. Bombycoid moths represent over 5000 species and exhibit wide diversity in flight styles, feeding habits, body sizes and wing morphology while maintaining similar component parts and actuation strategies [12,21]. Hawkmoths (family: Sphingidae) have evolved higher frequency wingbeats and smaller, high aspect ratio wings. Many hawkmoths perform impressively agile hover-feeding behaviours, using their long proboscides to consume nectar while matching the flower’s position mid-air [22]. Wild silkmoths (family: Saturniidae) generate larger amplitude, lower frequency wingbeats with lower aspect ratio wings compared with hawkmoths and lack functional mouth parts as adults. Some also possess characteristic colour patterns and long wing tails and have evolved a more erratic flight style to evade predators [23–25].

Using bombycoids as a model clade, we set out to answer two related questions:

— Do individual components of the hawk- and silkmoth flight system relevant to resonance (i.e. stiffness, wing hinge transmission and wing inertia) scale with wingbeat frequency in accordance with resonant tuning? The resonant frequency of a spring-mass damper typically increases with the square root of system stiffness and decreases with the inverse of the square root of inertia. Therefore, we predict these relationships for stiffness and inertia in moths, in agreement with resonance tuning. In particular, we predict the effects of stiffness and inertia to be strong enough to compensate for the higher wing hinge transmission ratio in smaller insects [20] that would in isolation reduce the resonant frequency with wingbeat frequency. Taken together, we hypothesize that variations in these individual components combine to result in a resonant frequency that matches wingbeat frequency in Bombycoidea to maintain generally favourable resonant mechanics regardless of wingbeat frequency.
— Do the resonant mechanics of hawk- and silkmoths reflect their different behavioural and energetic requirements? Given the stark clade-specific differences in wing morphology, kinematics and feeding habits, we expect silkmoths to have more aerodynamically efficient resonant properties than hawkmoths, reflecting their nutrient-limited adult life stage. We predict silkmoths to have a lower N than hawkmoths, resulting in a shallower resonance curve and providing them with a buffer of favourable frequencies around resonance at which to flap. As an alternative, silkmoths' low N may preclude any meaningful energetic benefit to operating at resonance. Conversely, hawkmoths’ agile hover-feeding behaviour leads us to predict they will be less constrained by precise matching of resonant and wingbeat frequencies and have a larger N. Deviation from resonance may enable frequency modulation at the expense of favourable energetics from perfectly resonant wingbeats. A larger N may allow them to return relatively more elastic energy than would be possible with a shallower resonance curve, even if operating off of resonance.

## 2. Methods

### (a) Spring-wing resonance modelling framework

We build upon a recent single degree-of-freedom, lumped-parameter dynamics model of a flapping insect [4,13]. Newton’s second law for a rotational system with an aerodynamic force that is proportional to the magnitude of velocity squared [4,8,26] is:


(2.1) $$I\;\overset{..}{\phi }\left(t\right)+\Gamma \left|\overset{˙}{\phi }\left(t\right)\right|\overset{˙}{\phi }\left(t\right)+\frac{k}{T^{2}}\phi \left(t\right)=\frac{F}{T}\sin\left(2\pi f_{wb}t\right)$$

Here, ϕ(t) is the dynamic variable and represents the time-varying wingstroke angle. This single equation is parameterized by the linear thorax stiffness k, transmission ratio T, inertia of wings and added mass I, aerodynamic damping coefficient Γ, wing beat frequency $f_{wb}$ and muscle forcing amplitude F. The absolute value in the damping term ensures that the direction of the damping force always opposes wing motion. Note that the elastic term has the coefficient $\frac{k}{T^{2}}$, which we refer to as the wing hinge rotational stiffness $k_{rot}$ . We also assume both k and T are independent of wing angle, which we justify in the following sections. Since this equation is a nonlinear second-order differential equation, we can numerically integrate it to solve it for the wing stroke angle ϕ(t). Doing so over a range of potential frequencies f yields a resonance curve (ϕ versus f), the maximum of which is the displacement (damped) resonant frequency $f_{res}$ . We are also concerned with the undamped resonant frequency, sometimes called the natural frequency $f_{nat}$ , which has the convenient closed form:


(2.2) $$f_{nat}=\frac{1}{2\pi }\sqrt{\frac{k}{T^{2}I}}$$

For lightly damped systems, $f_{res}$ and $f_{nat}$ are very close to one another, so their distinction is not important. However, since flapping insects are heavily damped by surrounding air, we compute both of these resonance frequencies which have slightly different physical meanings. $f_{res}$ is the maximum of the displacement versus frequency curve and is, therefore, indicative of the largest realizable flapping amplitude for some constant muscle force amplitude. $f_{nat}$ is the maximum of the velocity versus resonance curve and is the frequency at which no negative work is required by the muscle to drive flapping. In later sections, we discuss the precise mechanical implications of each frequency for insect flight. We detail the measurement procedure or computation of each parameter in the following sections.

### (b) Animals

Live specimens from 10 species of Bombycoid moths were used in this study. Hawkmoth species used were *Manduca sexta, Smerinthus cerisyi, Hyles lineata, Hemaris diffinis* and *Sphinx chersis*. Silkmoth species used were *Actias luna, Automeris io, Antheraea polyphemus, Hyalophora cecropia* and *Citheronia regalis*. Since thorax materials testing requires the animal to be deceased, attempts were made to record muscle strain data from live animals before thorax stiffness experiments.

### (c) Thorax stiffness measurements

We roughly followed the methods of precedent work with minor adjustments [4,17]. Briefly, we deformed moth thoraces over a range of frequencies at 9% strain peak-to-peak, and extracted stiffness at wingbeat frequency for each species, ignoring internal losses in the thorax due to structural damping and active muscle stiffness. See supplementary information for a detailed description of stiffness measurements.

### (d) Muscle strain measurements

Briefly, moths were tethered ventrally and positioned beneath a high-speed video camera. The moth's abdomen was removed and the middle part of the metathorax was partially dissected away, exposing the posterior phragma, the attachment point for the main downstroke muscles (DLMs). A white paint pen was used to mark muscle attachment points on each side of the animal. The moth was stimulated to flap and recorded from a dorsal view to capture muscle displacements and a front view to capture wingbeat amplitude.

We computed transmission ratio T directly from the phragma displacement $d_{max}$ and front-view wingstroke amplitude $\phi_{tethered}$ measurements in each moth individually.


(2.3) $$T=\frac{\phi_{tethered}}{d_{max}}$$

We defined the operating length $L_{op}$ of the thorax as the mean strain across all digitized wingbeats. We then used T, $L_{op}$ and free-flight wingbeat amplitude $\phi_{o}$ to calculate a muscle strain likely to be generated in free-flight by the following equation:


(2.4) $$\varepsilon =\;\frac{\phi_{o}}{TL_{op}}$$

See supplementary material for an extended description of transmission and strain measurements.

### (e) Inertia and aerodynamic damping calculation

We leveraged an existing wing morphometric dataset to calculate inertial and aerodynamic parameters for each moth species [21]. Specific imaging and digitization methods are identical to those in Aiello *et al*. 2021 [21]. For *H. cecropia, H. diffinis, S. cerisyi* and *S. chersis*, we used wing shape data from a species in the same genus: *Hyalophora euryalus, Hemaris thetis* and *Hemaris thysbe, Smerinthus ophthalmica* and *Smerinthus jamaicensis,* and *Sphinx kalmiae*. In the above cases, distinctions in wing morphology between species in the same genus were minimal and should not meaningfully affect our results.

With this measure of inertia, we computed the fraction of inertial work offset by elastic energy storage. Integrating the elastic and inertial terms of equation (2.1) over a quarter-stroke yields the work contributions $\frac{k}{T^{2}}\phi_{o}^{2}$ and $I\phi_{o}^{2}{\left(2\pi f_{wb}\right)}^{2}$. We considered only a quarter-stroke since the symmetry of a sinusoidal wingstroke means that the calculation will be the same. Taking the quotient of these expressions gives us an estimate of the amount of energy returned by the spring relative to its potential maximum benefit.


(2.7) $$\hat{K}=\frac{k}{T^{2}I{\left(2\pi f_{wb}\right)}^{2}}$$

Note that $\hat{K}$ also has the convenient interpretation as the squared ratio of the natural frequency and wingbeat frequency ($\hat{K}=f_{nat}^{2}/f_{wb}^{2}$). This form highlights the energetic significance of $f_{nat}$ for insects, as it is the frequency at which inertial power offset is 100%, thus requiring no negative work from the musculature. See supplementary information for a further detailed description of calculation of inertia and aerodynamic damping.

### (f) Moth free-flight wing beat frequency and amplitude

We leveraged existing wind tunnel free-flight videos of moths, which were collected and digitized as described by Aiello *et al*. 2021 [12]. Wing strokes from multiple individuals were averaged to yield a species-specific frequency and stroke plane sweep-angle amplitude. For *H. cecropia, S. chersis, H. diffinis* and *S. cerisyi*, we used free-flight data from similarly sized and closely related species of the same genus *H. euryalus, S. kalmiae, H. thysbe* and *S. ophthalmica* instead. Time-varying angle-of-attack data for each species was also used to compute a wingstroke-averaged species-specific drag coefficient.

### (g) Simulation

Using the parameters from each species, we simulated a frequency sweep from 1 to 100 Hz using species-averaged parameter values for *k*, *T*, *I* and Γ. Muscle forcing amplitude F was computed for each species by selecting the value that results in a wingbeat amplitude that matches free-flight experiments for a simulated insect driven at its free-flight wing beat frequency. This method accounts for the fact that *in vitro* muscle physiology experiments on moths have resulted in force estimates that are over an order of magnitude lower than what would be required to sustain flight [4,27,28]. Resonant frequency $f_{res}$ was computed as the frequency at which maximum steady-state wingbeat amplitude occurs.

### (h) The Weis-Fogh number and aerodynamic efficiency

The Weis-Fogh number was originally defined as the ratio of peak inertial to peak aerodynamic torque over a wing stroke, given by the following equation:


(2.9) $$N=\frac{I}{\Gamma \phi_{o}}$$

Using this equation to compute N across species relies upon a heavily simplified aerodynamic model with a constant damping parameter. As a way to incorporate slightly more aerodynamic realism, we computed N using wingbeat-averaged aerodynamic and inertial power computations from a recent blade-element model [12]. See electronic supplementary material for a derivation of this equation.


(2.10) $$N=\frac{4}{3}\frac{{\overset{-}{P}}_{inertial}}{{\overset{-}{P}}_{aero}}$$

Weis-Fogh originally defined the aerodynamic efficiency of a flapping insect as the fraction of total work required for flight taken up by aerodynamic costs [8]. Aerodynamic costs represent the ‘useful’ work that contributes to supporting body weight, while inertial and elastic costs are necessary to actuate flight but do not aid in body weight support. We introduce two non-dimensionalizations to make the computation of these energy costs easier [13]. First, we non-dimensionalized the wing angle in equation (2.1) by the wing stroke amplitude such that $\hat{\phi }(t)=\phi (t)/\phi_{o}$. We then non-dimensionalized each torque in equation (2.1) by the peak aerodynamic torque at the midstroke, resulting in the following non-dimensional torques as a function of non-dimensional wing angle $\hat{\phi }$.


(2.11) $${\hat{\tau }}_{aero}(t)=1-(\hat{\phi }(t){)}^{2}$$

(2.12) $${\hat{\tau }}_{inertial}(t)=-N\hat{\phi }(t)$$

(2.13) $${\hat{\tau }}_{elastic}(t)=\hat{K}N\hat{\phi }(t)$$

We can then integrate each torque to yield the work associated with each energy cost. We only consider positive work, as negative work done by muscles incurs significantly less metabolic energy cost and is often modelled as negligible [9,29]. Choosing the bounds of integration such that only positive work is considered (see electronic supplementary material, S1), we can compute aerodynamic efficiency by the following equation:


(2.14) $$\eta =(100)\frac{\int_{+}{\hat{\tau }}_{aero}(t)d\hat{\phi }}{\int_{+}{\hat{\tau }}_{inertial}(t)+{\hat{\tau }}_{elastic}(t)+{\hat{\tau }}_{aero}d\hat{\phi }}$$

This definition of aerodynamic efficiency is distinct from any notion of metabolic efficiency, which we are not directly assessing in the current work.

### (i) Statistics

We use two-sample *t*-tests to compare clade-dependent differences (grouped as hawkmoths and silkmoths) in resonant mechanical properties with a significance threshold of 0.05. For continuous data, we perform linear regressions and illustrate a line and confidence intervals only if we find a significant relationship. We report $r^{2}$ values as the squared Pearson correlation coefficient and p values for each statistical test in parentheses. In addition, we perform phylogenetic least squares (PGLS) to confirm that any apparent trend cannot be attributed to phylogenetic distance between species alone. We implement PGLS in R (v. 4.2.1) with the phytools (v. 2.1-1) and caper (v. 1.0.3) packages [30].

## 3. Results

### (a) Thorax stiffness does not increase with wingbeat frequency

**Figure 2.**
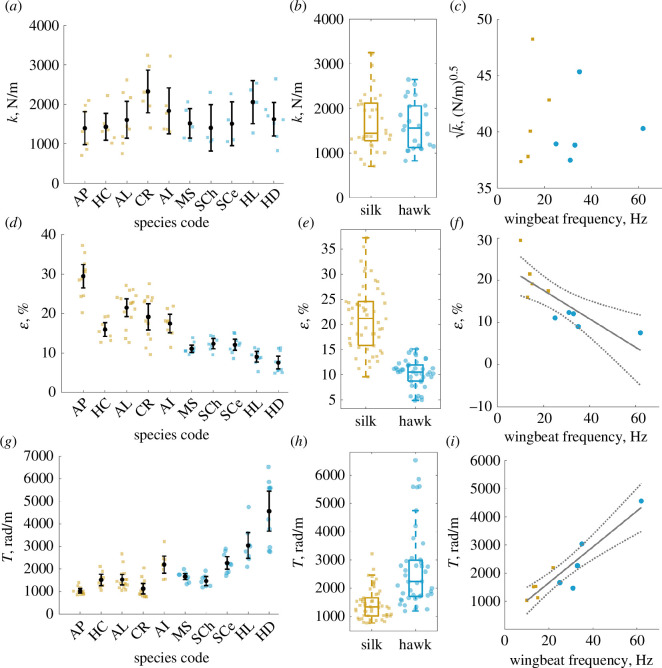
Stiffness does not show a distinct pattern of variation across species. Each marker represents a different individual, with orange squares corresponding to silkmoths and blue circles corresponding to hawkmoths. Species are ordered in increasing wingbeat frequency. (*a*) Black circles are species means, and error bars show 95% confidence intervals of the mean. (*b*) Stiffness does not differ between clades within Bombycoidea (p=0.615). Boxplots show median, quartiles and 1.5×(IQR). (*c*) There is no significant relationship between $\sqrt{k}$ and wingbeat frequency (linear regression$:\;r^{2}<0.01,\;p=0.971$). (*d*) Thoracic strain varies between species. For (*d,e*) and (*g,h*), each marker corresponds to a single flight bout from a single individual. (*e*). Silkmoths have higher average strains than hawkmoths (*p<0.001). (*f*) Strain varies with wingbeat frequency in an inverse fashion (linear regression: *r*^2^ = 0.624, *p = 0.007). (*g*) Transmission ratio varies strongly with species. (*h*) Hawkmoths have higher transmission ratios than silkmoths (*p<0.001). (*i*) Transmission ratio varies in a strong positive linear fashion with wingbeat frequency (linear regression: *r*^2^ = 0.850, *p < 0.001).

To identify how components of the flight system contribute to resonant mechanics across moths of varying wingbeat frequency, we measured stiffness, transmission and inertia comparatively in hawk- and silkmoths. We performed dynamic materials testing over a range of frequencies to measure thorax stiffness, extracting the stiffness of the thorax at wingbeat frequency for each moth (figure 2*a*). We found that stiffness does not vary across the nearly order of magnitude of wingbeat frequency variation captured in our species sampling, and there are no detectable differences between hawkmoths and silkmoths when grouped by clade (figure 2*b*). Substantial intersubject variation is persistent across species (figure 2*a*). Since the resonant frequency of a spring-mass damper is proportional to the square root of stiffness, we expect $\sqrt{k}$ to scale with the wingbeat frequency of an insect flapping at resonance. However, we could not detect a statistically significant relationship between wingbeat frequency and $\sqrt{k}$ , including when controlling for phylogenetic relatedness (figure 2*c*, electronic supplementary material, table S2). Stiffness measurements were lower on average than some previous measurements, which we attribute to the fabrication of three-dimensional printed mounts that better match the DLM attachment surfaces, thus resulting in more realistic deformations [11,31]. The current results are in close agreement with recent independent measurements from *Manduca*, and within the range of previous measurements with intact metathorax, thus confirming that the metathorax is not significantly responsible for energy storage [11,31,32]. Regardless, stiffness does not coevolve with wingbeat frequency.

### (b) Muscle strain decreases and transmission ratio increases with wingbeat frequency

We next examined whether muscle strain and transmission ratio vary with wingbeat frequency in a way that facilitates resonance tuning. Muscle strain amplitude varied inversely with wingbeat frequency, with over 20% difference between the lowest and highest frequency animals (figure 2*d,f* ). *A. polyphemus* exhibited the largest muscle strain amplitude, with individuals exceeding 30%, a large peak-to-peak strain for a cyclic movement at around 10 Hz. Other slow-flapping silkmoths we measured only exhibited strains of 16–25% (figure 2*d*), suggesting *A. polyphemus* has muscles capable of particularly high strain. On average, silkmoths exhibited over twofold larger strains than hawkmoths (figure 2*e*). Transmission ratio is a composite quantity that takes muscle strain, thorax length and wingbeat amplitude into account. We find transmission scales roughly linearly with wingbeat frequency (electronic supplementary material, table S2), such that higher frequency moths have a larger transmission ratio (figure 2*g–i*). This follows directly from the muscle strain result because muscle strain and transmission ratio are inversely proportional. Between our fastest and slowest moths, transmission ratio varied by a factor of 5 (figure 2*i*).

### (c) Hawkmoths and silkmoths both offset inertial power costs with elastic energy storage

**Figure 3.**
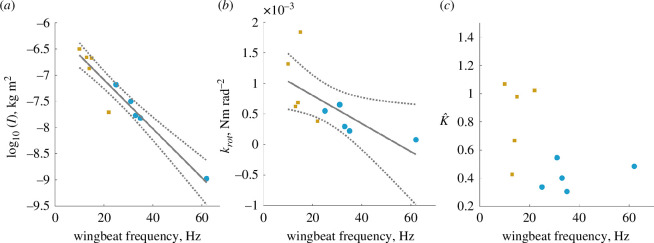
(*a*) The logarithm of wing inertia decreases sharply and nonlinearly with frequency (regression: $r^{2}=0.925$, *p* < 0.001*). (*b*) Linear regression of rotational stiffness versus wingbeat frequency shows a weak negative relationship ($r^{2}=0.442$, $p=0.036^{*}$), which persists when controlling for phylogeny. (*c*) Moth species offset widely varying proportions of their inertial power costs with elastic energy storage and return, although on average the energetic benefit is significant, approximately 57±28%. There is no significant relationship between wingbeat frequency and $\hat{K}$ (p=0.151). In all plots, linear regression lines are shown in solid grey, with 95% confidence intervals shown in dotted grey. All individual points correspond to species-average values.

Combining thorax stiffness and transmission ratio into the wing rotational stiffness ($k_{rot}$), we find a weak inverse relationship between rotational stiffness and wingbeat frequency (figure 3*b*) that persists under PGLS (figure 3, electronic supplementary material, table S2). This result is contrary to common intuition that faster oscillators are stiffer, as well as some previous scaling predictions for rotational stiffness as a function of body size [33]. Wing inertia falls off sharply and in a nonlinear fashion with wingbeat frequency, ranging over 2.5 orders of magnitude (figure 3*a*). This decrease is much stronger than what would be expected from the −0.5 exponent suggested by equation (2.2). From equation (2.7), we compute the fraction of inertial power offset by elastic energy storage from rotational stiffness and wing inertia. Surprisingly, these two quantities combine to result in no discernable relationship between inertial power offset and wingbeat frequency (figure 3*c*, electronic supplementary material, table S2). Both hawk- and silkmoth thoraces return substantial energy from cycle to cycle, with the lowest $\hat{K}$ hawkmoth still offsetting 20% of its inertial costs. The slowest moth we tested, *A. polyphemus*, has a $\hat{K}\approx 1.07$, indicating that its thorax elasticity is slightly overtuned with respect to its inertial costs (figure 3*c*).

### (d) Bombycoid resonant mechanics reflect behavioural and energetic requirements

**Figure 4.**
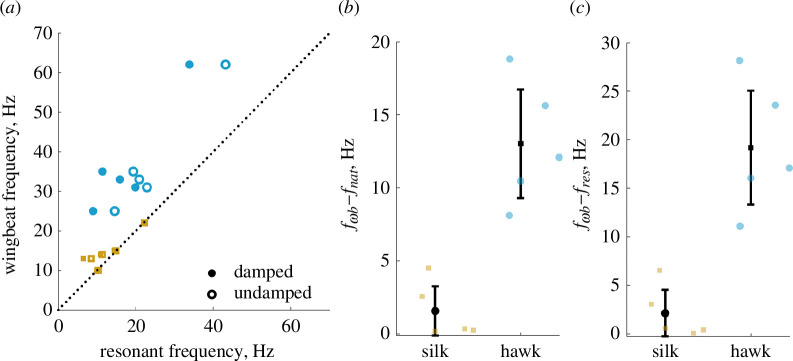
(*a*) Both undamped ($f_{nat}$) and damped ($f_{res}$) resonance frequencies of moths lie above the equivalency line, indicating that Bombycoid moths are generally supra-resonant. (*b,c*) Difference between wingbeat frequency and resonance frequency (undamped and damped) is larger in hawkmoths than in silkmoths.

Combining measurements of thorax properties, muscle strain, wing morphology and free-flight kinematics, we can compute resonance frequencies for each species of moth from our spring-wing model equations (2.1) and (2.2). We find a correlation between moth wingbeat frequency and both undamped ($f_{nat}$) (linear regression $r^{2}=0.890,\;p<0.001^{*};\;PGLS:\;r^{2}=0.887,\;p<0.001^{*}$) and damped ($f_{res}$) (linear regression: $r^{2}=0.629,p=0.006^{*};PGLS:r^{2}=0.635,p=0.006^{*}$) resonance frequencies (figure 4*a*). In general, we find that undamped resonance frequency is a good predictor of wingbeat frequency across species, as evidenced by most species lying very close to the equivalency line in figure 4*a*. Damped resonances lie farther from wingbeat frequencies because consideration of aerodynamic damping reduces the system resonant frequency further. Regardless of which resonance frequency is being considered, hawkmoths lie at least 10 Hz farther from resonance on average than silkmoths (damped $f_{res}$ : p<0.001, undamped $f_{nat}$ : p<0.001) (figure 4*b,c*).

**Figure 5.**
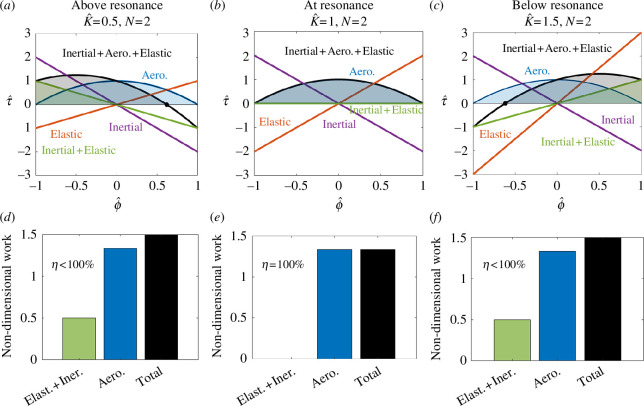
Non-dimensional torques and work at three different resonant conditions. (*a,b*) Above (faster than) resonance, inertial torque exceeds elastic torque, creating a net positive work cost during the first half of each halfstroke (green shaded area and bar). As such, aerodynamic efficiency is below 100%. (*c,d*) At resonance, inertial and elastic torques cancel exactly at every point during the wing stroke, so the only source of net positive work is due to aerodynamics. As such, aerodynamic efficiency is 100%. (*e,f*). Below resonance, elastic torques exceed inertial torques resulting in a net positive work cost during the second half of each halfstroke (green shaded area and bar). This results in the same efficiency as in the above resonance case.

While operation close to a resonant peak is generally indicative of efficiency, resonant frequency alone is not enough to quantitatively evaluate a moth’s aerodynamic efficiency. This is because efficiency also depends on the shape of an insect’s resonance curve, which can be captured by the Weis-Fogh number N. An insect with a wider resonance curve (lower N) will incur lesser relative energetic penalties for operating off of resonance. To evaluate contributions of resonance curve shape and distance from the resonant peak to overall animal performance, we compute each moth species' aerodynamic efficiency. Weis-Fogh originally defined this quantity as the ratio of aerodynamic work to total work required by an insect over a wing stroke [8]. Using equation (2.1), we can write expressions for non-dimensional torques about the wing hinge as a function of non-dimensional wing angle. The energy cost associated with each torque is then represented by the integral of that torque with respect to the wing angle. We assume that only positive work contributions require significant metabolic energy and compute aerodynamic efficiency as the ratio of blue and grey areas in figure 5*a–c* (a more detailed mathematical description of this calculation is in the electronic supplementary material). We diagrammatically show the integrals of equation (2.11), (2.12) and (2.13) in figure 5 in three resonant regimes: above, equal to and below undamped resonance ($f_{nat})$. Above and below resonance, inertial torque exceeds elastic torque during a portion of each halfstroke, resulting in a positive work cost (green shaded area and bars in figure 5*a,d*,*c*,*f*) that must be supplied by musculature. At resonance, inertial and elastic torque cancel exactly at all $\hat{\phi }$ so that the only mechanical work required of the musculature is due to aerodynamics (figure 5*b,e*).

**Figure 6.**
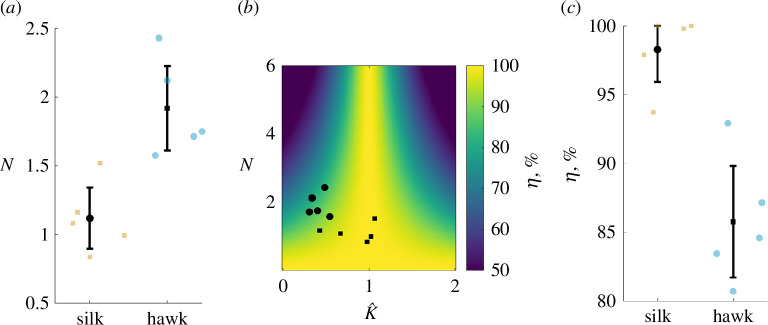
(*a*) Weis-Fogh number (N) is larger in hawkmoths than silkmoths (p=0.003). (*b*) Aerodynamic efficiency space for resonant flappers depends on N and $\hat{K}$. Hawkmoths (dots) and silkmoths (squares) cluster differently in this space. (*c*) Combining resonant frequency and the Weis-Fogh number for each moth species reveals that silkmoths have higher aerodynamic efficiencies on average than hawkmoths (p<0.001).

Having established a measure of aerodynamic efficiency that depends on N, we use equation (2.10) to compute N comparatively. We estimate that N values for our moths lie between 1 and 5, in agreement with previous estimates for similar insects (figure 6*a*). Importantly, each species had 1<N<5 indicating potential for elastic energy offset of inertial costs, but with a relatively shallow resonance curve, especially when compared with engineered oscillators, which have quality factors that far exceed 10 [34]. We find that hawkmoths have a larger N on average than silkmoths (figure 6*a*). When we calculate aerodynamic efficiency using equation (2.14), the resulting space depends only on two parameters, N and $\hat{K}$ (figure 6*b*). A maximum occurs at $\hat{K}=1$ , which corresponds to operation at undamped resonance ($f_{nat}$) (figure 5*d*). Efficiency decreases with increasing N, with this effect being steeper farther away from resonance. A smaller N implies a larger relative aerodynamic cost, thus a greater fraction of the insect’s per-wingstroke energy budget is being devoted to useful aerodynamic work. When we place hawkmoths and silkmoths in this efficiency space, we find that they cluster differently based on their differing $\hat{K}$ and N values. We find silkmoths have 10% higher aerodynamic efficiency on average than hawkmoths (p<0.001), mostly due to their lower average N(p=0.003) (figure 6*c*). Hawkmoths’ higher N places them in a location in the space with a steep efficiency gradient. Therefore, their supra-resonant wingbeats incur a larger efficiency penalty than would be the case at a lower N.

## 4. Discussion

Our first objective in this study was to understand whether components of the flight system vary with wingbeat frequency in a way that facilitates or works against resonance tuning in moths. We find strong support for our hypothesis that wingbeat frequency scales with resonant frequency, but not because of the expected scaling of stiffness. Our results demonstrate no clear relationship between thorax stiffness and wingbeat frequency (figure 2*a–c*), but a positive linear relationship between transmission and wingbeat frequency (figure 2*g–i*). Combined, these trends result in a wing hinge rotational stiffness that decreases with wingbeat frequency (figure 3*b*). This trend alone is contrary to the expected positive scaling of rotational stiffness to result in resonant tuning. However, due to the sharply nonlinear inverse scaling of wing inertia with wingbeat frequency (figure 3*a*), resonance frequency scales linearly with wingbeat frequency (figure 4*a*). Moreover, in most cases, wingbeat frequencies tend to be offset such that they exceed the resonant frequency (figure 4*a–c*).

Second, we aimed to link clade-specific variation in resonant mechanics (i.e. proximity to resonance and N) to clade-specific behavioural and energetic requirements. We developed a new measure of aerodynamic efficiency that considers both N and $\hat{K}$ based upon the original work of Weis-Fogh. We found that silkmoths operate very close to undamped resonance ($f_{nat}$) (figure 4*a–c*) and have a lower N on average than hawkmoths (figure 6*a*), which results in a shallower resonance curve and higher aerodynamic efficiency (figure 6*a,c*). In contrast, hawkmoths operate farther from resonance than silkmoths and their larger N places them in a location in efficiency space with a steep gradient. Thus, N appears to be an important determinant of aerodynamic efficiency, dictating the degree to which off-resonance behaviour incurs an energetic penalty. Indeed, in this region of efficiency space, a difference in N by 1 can reduce efficiency by nearly 20% (figure 6*b*). This result is particularly interesting since silkmoths do not feed as adults, as they lack functional mouthparts [23]. As such, they are significantly more nutrient limited over their life history, which may have led to selection for higher aerodynamic efficiency as a means of increasing their viable reproductive period [12]. Hawkmoths, instead, may take advantage of their above-resonance wingbeats to more easily modulate wingbeat frequency to manoeuvre while tracking flowers [6], while maintaining sustainable energetics by virtue of a sharper resonance curve.

### (a) Constraints on wingbeat frequency-scaling of thorax and wing properties

Our transmission ratio scaling results (figure 2*g–i*) illustrate how muscle physiology may place a fundamental constraint on resonant mechanics. It is generally the case that faster movements are generated by smaller muscle strains. This allows muscles to operate on a narrow, more favourable location on their length-tension curve and produce higher forces at lower velocities, thus producing larger amounts of power [20]. As such, our data suggests that these inherent muscle properties that mandate an increasing transmission ratio with frequency may be a more important constraint on thorax mechanics than any potential efficiency benefit from a stiffer thorax. Indeed, both hawkmoths and silkmoths already offset at least 50% of their inertial power costs with elastic energy storage over a range of wingbeat frequencies (figure 3*c*), suggesting that there might not be a large advantage to tuning thorax stiffness more precisely.

Biomechanical constraints imposed by thorax geometry and material may provide additional context for the invariance of thorax stiffness in moths. The thoracic shell is a highly intricate structure, with a shape that localizes strain energy in certain regions of the exoskeleton [11,35]. Shape, rather than material, likely determines bulk stiffness similar to how curvature affects stiffness of the human foot arch [36]. Despite variation in thorax shape and size between moths of different species, we show that bulk stiffness remains the same (figure 2*a–c*), which is ultimately the stiffness that muscles encounter when actuating flight. As such, it may be very difficult to precisely tune thorax stiffness over evolutionary time without interfering with its functionality or structural integrity. In addition, it seems inefficient to modulate resonant frequency via thorax stiffness. Since resonant frequency is proportional only to the square root of stiffness, very large stiffness changes would be necessary to manifest in substantial resonant frequency variation across species. Such large stiffness variation may have negative consequences for the animal, like prohibitively high impedance for the flight muscles during take off. This does not preclude the hypothesized action of steering muscles to modulate stiffness within an animal over short timescales, as frequency modulation ranges for insects like *Manduca* are small compared with interspecies frequency differences [6]. Finally, since aerodynamic and inertial power requirements scale sharply with frequency, a large amount of flight muscle in proportion to body size is required to drive flight in faster organisms [37]. A need to pack as much power muscle as possible into the thorax may restrict any geometry-driven stiffness variation in the thorax over evolutionary time. Comparative analysis of muscle and thorax morphology across this group may shed light on how muscle constrains thorax properties.

Despite stiffness being nearly invariant and transmission ratio increasing with wingbeat frequency, we still find a proportional relationship between resonant frequency and wingbeat frequency. This is reflective of the importance of wing inertia in dictating an insect’s resonant mechanics. Wing inertia-frequency scaling is strong enough to compensate for the increase in transmission ratio that is required to generate high-frequency wingbeats. Wing inertia in moths is a combination of wing mass and shape, the latter being a trait that varies extensively across lepidopterans [21]. Wing mass distribution based upon tapering or venation patterns may be an orthogonal axis by which wing inertia can be modulated over evolutionary time. We do not explicitly consider this variation in the present work, which may increase resonant frequencies of insects that pack significant mass closer to their wing hinge. We propose that maintaining favourable resonant mechanics is an additional pressure in the evolutionary tuning of moth wing shape and size, counteracting the effects of increasing transmission ratio with wingbeat frequency. In cold-hardy geometrid moths, low wing inertia has been shown to be the primary driver of reduced flight power costs that enable flight at extremely low body temperatures [38]. Thus, wing inertia appears to be a particularly efficient ‘knob’ by which to tune flight energetics over evolutionary time in moths.

### (b) Multiple resonant peaks, nonlinearity and band-type resonance

We apply a simple model of a single resonance peak that is grounded in recent detailed work on *Manduca* [4]. However, any conclusion that an animal is or is not at resonance depends on the precise definition of resonance being used. We provide comparisons of moth wingbeat frequency to both damped ($f_{res}$) and undamped ($f_{nat}$) resonance but note that the aerodynamic efficiency depends only on proximity to undamped resonance $\left(\hat{K}\right)$. Undamped resonance ($f_{nat}$) is the frequency at which no dissipation is required by the muscle to drive flight, and represents the frequency at which inertial and elastic costs are instantaneously balanced at every point in a wingstroke (figure 5*b,e*). Most recent studies of insect resonance have been concerned with damped resonance, which maximizes wingbeat amplitude for a given input muscle force. Thus, operating at damped resonance may also be ‘optimal’, just by the different criterion of maximal wingbeat amplitude as opposed to aerodynamic efficiency. Our work suggests that investigation of multiple resonant frequencies and efficiency metrics may be necessary to fully contextualize an organisms' preferred movement frequency.

Indeed, recent work by Pons *et al*. has demonstrated multiple distinct resonance frequencies that exist in the presence of thoracic nonlinearities in the flight motor [14,39]. Such nonlinearities in hawkmoths are likely small [11], but may become more significant at the high strains experienced by silkmoth thoraces. Similarly, we do not explicitly include the effects of active muscle stiffness. While muscle itself can store and return energy [40], active muscle stiffness is low compared with thorax stiffness in *Manduca* [27] and thus, does not contribute highly to resonance. This is likely not the case in some small flying insects like flies, where muscle stiffness is the dominant stiffness in the thorax [18]. We do not know whether active muscle stiffness introduces a more significant nonlinearity in high-strain silkmoths. In addition, we do not explicitly model series elasticity in the wing hinge on the grounds that such effects are likely extremely small in *Manduca* [4,41]. Inclusion of substantial series elasticity in the wing hinge would primarily serve to widen the resonant peak, increasing the allowable frequencies of operation with minimal loss of efficiency [14,39]. In summary of all available evidence, wing hinge compliance and thorax nonlinearity likely do not strongly influence resonance in bombycoids, but are increasingly important at the scale of *Drosophila* and smaller.

### (c) Insect flight resonance beyond moths

We focus on Bombycoidea as a model clade for studying resonant mechanics in closely related species against the backdrop of significant wing morphological and behavioural diversity. However, we highlight a number of general principles that may apply broadly to other groups of insects and areas for further comparative study. Increasing transmission ratio with wingbeat frequency is a general feature of flapping systems [20], and imposes a constraint on resonance in any clade that varies in wingbeat frequency. Any group of insects that vary in size will have to work against transmission ratio scaling to achieve resonance tuning. We show that in bombycoids, wing inertia scaling is strong enough to overcome the transmission ratio (figures 2*i*, 3*a* and 4a), but in other clades with more geometrically similar wings across body sizes, this may not be the case. Taking advantage of large intraspecific variation in insects like bees and studying resonant properties at an individual level may shed light on whether inertia is the main driver of resonance tuning more generally [42].

Many clades of insects such as Coleoptera, Hymenoptera and Diptera do not control their wings with a time-periodic nervous system signal but instead, actuate flight via antagonistic stretch-activated flight muscles [43,44]. In so-called asynchronous insects, wingbeat frequency is emergent so they are often thought to be flapping at resonance by definition and have little neural control over their wingbeat frequency. Even so, bumblebees are capable of buzzing at multiple discrete frequencies that correspond to different behaviours such as thermogenesis, communication, buzz pollination and flight [45]. Unlike hawkmoths, which can modulate frequency by neural activity, bees most likely achieve different wingbeat frequencies by modulating resonant properties of their thorax with steering muscles or transmission ratio via changing wing deployment. If insects like bees are operating close to resonance, our results suggest that they can accommodate a larger N while maintaining higher aerodynamic efficiency. Estimates from bees suggest N>6, which would incur a large efficiency loss if they were sufficiently off of resonance (figure 6*b*). Alternatively, bees may endure this loss while maintaining moderate elastic energy storage in order to modulate frequency more widely, suggesting that frequency control may be at least as important as power and efficiency in insect flight.

Our work highlights the multivariate, often conflicting demands on the musculoskeletal systems of animals that use fast, oscillatory locomotion. Unlike commonly studied spring-driven ballistic animal movements [46], flapping insects must negotiate challenges associated with power production, dissipation and control on a wingstroke-to-wingstroke basis. Similarly, myriad terrestrial animals take advantage of resonant mechanics to improve locomotor efficiency by cyclically storing energy in tendons or apodemes [3]. Resonance tuning, while an elegant explanation for insects' preferred frequency of movement, requires a particular combination of thoracic spring, muscle physiology, wing transmission and wing shape properties. Each of these components serves multiple functions and may vary counter to the expectation from resonance, such as in the case of wing transmission. In the case of bombycoid moths, wing inertia appears to be the primary knob by which resonance tuning is achieved. But even though resonance frequency scales with wingbeat frequency, bombycoids still operate somewhat off of resonance, both with and without damping. Trade-offs between proximity to resonance and N allow both hawkmoths and silkmoths to fly with feasible aerodynamic efficiency while operating within the constraints imposed by their muscle, thorax morphology and behaviour.

## Data Availability

Data can be accessed at the Georgia Tech digital repository [47]. Supplementary material is available online [48].
